# An Exploration of the Role of Acetamidinium Substitution in Methylammonium Lead Iodide Perovskites

**DOI:** 10.1002/cphc.202500259

**Published:** 2025-11-23

**Authors:** Fernando Brondani Minussi, Rafaela Coutinho de Oliveira Santos, Marco Antonio de Mello Teixeira, Rogério Marcos Silva, Eudes Borges Araújo

**Affiliations:** ^1^ Department of Physics and Chemistry São Paulo State University Ilha Solteira SP 15385‐000 Brazil; ^2^ Department of Electrical Engineering São Paulo State University Ilha Solteira SP 15385‐000 Brazil

**Keywords:** acetamidinium, electrical properties, halide perovskites, methylammonium lead iodide, stability and degradation

## Abstract

Halide perovskite (HP) versatility for optoelectronic and electrochemical applications is mainly due to their ability to engineer cation mixtures at the A‐site within the ABX_3_ stoichiometry. Acetamidinium (AC^+^) is a common cation used in these mixed compositions, but its effects on the material's properties have not been addressed in detail. In this work, prototypical methylammonium lead iodide (MAPbI_3_) compositions partially substituted with AC^+^ are synthesized and analyzed for structural, electrical, optoelectronic, and stability properties. Results reveal a solubility limit of around 10% AC^+^, lower than encountered in the literature, with slight effects on the phase transition temperatures. As expected, substitution with AC^+^ significantly reduces electronic conductivity and *I*–*V* hysteresis but only marginally increases the bandgap energy. Contrary to literature results, light‐accelerated degradation tests show that AC^+^ incorporation does not significantly enhance the materials’ stability. Among several reasons, this might be related to weak interactions between AC^+^ cations and the inorganic framework. This study establishes the effects of AC^+^ substitution in halide perovskites and provides insights into optimizing A‐site compositions for optoelectronic and electrochemical applications.

## Graphical abstract

Acetamidinium (AC^+^) is one of the many organic cations that can be used as a partial substituent to modulate the properties in halide perovskite materials and devices. However, not much is known about how it affects some fundamental characteristics of the host materials. Here, it is shown that AC^+^ changes the prototypical MAPbI_3_, but intriguingly not much.

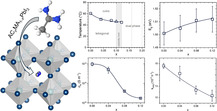

## 1 Introduction

Lead halide perovskites have emerged as a class of materials with significant technological potential, particularly in optoelectronic devices and photovoltaics, offering a more efficient alternative to traditional silicon‐based solar cells.^[^ ^1^, ^2^ ^]^ Their chemical composition follows the general formula ABX_3_, characterized by a 3D arrangement of edge‐sharing [BX_6_] octahedra.^[^ ^3^ ^]^ For example, the prototypical methylammonium lead iodide (MAPbI_3_) perovskite has well‐known exceptional performance in photovoltaic applications.^[^ ^4^ ^]^ In MAPbI_3_, the A‐site is occupied by the methylammonium cation (CH_3_NH_3_^+^ or MA^+^), the B‐site by a divalent lead cation (Pb^2+^), and the X‐site by an iodine anion (I^−^). Despite their high efficiency, halide perovskites face challenges related to degradation, arising from their interactions with light, humidity, and heat, which can compromise their performance in practical applications.^[^ ^5^, ^6^ ^]^

Studies have explored the incorporation of mixed cations at the A‐site in the synthesis of perovskites, aiming to enhance stability and investigate the resulting changes in the physicochemical properties of these materials.^[^ ^7^, ^8^ ^]^ The instability of perovskites is a widely discussed issue in the scientific literature, with its origins attributed to several factors, including alterations in crystal structure due to temperature fluctuations,^[^ ^9^ ^]^ degradation from exposure to environmental factors such as oxygen, humidity, and heat,^[^ ^10^, ^11^ ^]^ as well as phase transformations that may lead to property changes detrimental to device performance.^[^ ^12^, ^13^, ^14^ ^]^ A key factor influencing the stability of the perovskite structure is the size of the A cation, as it directly impacts the structural symmetry and, consequently, the physical and chemical properties of the material.^[^ ^5^, ^15^ ^]^ Significant progress has been made by using various A cations to modulate specific properties in lead halide perovskites, such as thermodynamic stabilization and ion migration driven by electrostatic interactions, among others.^[^ ^16^ ^]^ However, the substituent cation size is not the sole chemical descriptor that governs the materials’ characteristics.^[^ ^16^, ^17^ ^]^

The literature highlights several cations used in the A‐site cation mixtures within the MAPbI_3_ system, including guanidinium ([C(NH_2_)_3_]^+^ or GA^+^), formamidinium ([HC(NH_2_)_2_]^+^ or FA^+^), ethylammonium (CH_3_CH_2_NH_3_^+^ or EA^+^), and acetamidinium ([CH_3_C(NH_2_)_2_]^+^ or AC^+^).^[^ ^16^ ^]^ Generally, these substituent cations have suitable ionic radii to keep tolerance factors within the perovskite phase ranges^[^ ^18^ ^]^ and can lead to enhanced stability due to increased configurational entropy.^[^ ^5^ ^]^ It was proposed that systems of the A_*x*_MA_1−*x*_PbI_3_ type have demonstrated a greater tendency to form single‐phase solid solutions when the substituting cation has more N—H bonds than the substituted MA^+^ cations.^[^ ^19^ ^]^ One important aspect in this matter is the fact that the existing studies in the AC_*x*_MA_1−*x*_PbI_3_ system report different substitution degrees above which AC^+^‐based secondary phases start to segregate, ranging from *x* = 0.15 to 0.50,^[^ ^20^, ^21^, ^22^, ^23^ ^]^ demonstrating a lack of consensus regarding the solubility limit of this cation in MAPbI_3_‐based compositions. Moreover, A‐site substitution is known to change the phase transition temperatures in halide perovskites;^[^ ^1^ ^]^ interestingly, the effects of AC^+^ cations have only been marginally studied in this context.^[^ ^17^ ^]^

Regarding the stability issues, with its greater number of N—H bonds than the MA^+^ cation, the AC^+^ cation may contribute to enhanced stability in halide perovskites composed of the AC_*x*_MA_1−*x*_PbI_3_ system.^[^ ^21^ ^]^ Studies have shown characteristics related to hydrogen interactions involving N—H and the iodine of the [PbI_6_] octahedra. These interactions establish strong electrostatic forces that stabilize the AC^+^ cation within the perovskite matrix.^[^ ^21^, ^24^ ^]^ Light degradation tests demonstrated that devices made of AC_0.1_MA_0.9_PbI_3_ thin films retained 70% of their efficiency after 480 h, showing greater stability than MAPbI_3_ solar cells.^[^ ^21^ ^]^ This indicates an improvement in the stability of the perovskite when exposed to light, heat, and humidity, suggesting that the AC^+^ cation may be a promising candidate for enhanced stability in perovskite‐based applications. Other studies compare the use of organic cations GA^+^ with AC^+^, suggesting that substitution in the MAPbI_3_ system leads to more stable structures due to hydrogen bonds within the inorganic framework. Notably, the MAPbI_3_ system with 15% AC^+^ substitution exhibited improved charge carrier mobility and a lower defect density, increasing power conversion efficiency.^[^ ^20^ ^]^ Additionally, results indicate better performance for perovskite‐based devices in the AC_*x*_MA_1−*x*_PbI_3_ system with only 3% AC^+^ substitution. On the other hand, with light‐accelerated degradation tests, it was recently found that substituting with 10% AC^+^ has minimal effect on the stability of MAPbI_3_‐based compositions compared to the substitution with 10% GA^+^.^[^ ^17^ ^]^ In this scenario, it must be pointed out that most studies were performed in thin films and devices, which may have additional substrate, surface, and microstructural factors that potentially affect the observed outcomes in perovskite devices.^[^ ^25^ ^]^

Given the conflicting information regarding the impact of the AC^+^ cation on the perovskite lattice, a detailed analysis of the properties of the AC_*x*_MA_1−*x*_PbI_3_ system with lower degrees of substitution is essential. More than that, a whole characterization in bulk AC_*x*_MA_1−*x*_PbI_3_ perovskites with systematically variable AC^+^ content is still missing, making it difficult to understand the real effects of this cation on the perovskite matrix and the observed properties. In this sense, the present work aims to investigate the structural, microstructural, thermal, and electrical characterization processes, as well as light‐accelerated degradation behavior, in the AC_*x*_MA_1−*x*_PbI_3_ halide perovskite system with AC^+^ contents from *x* = 0.00 to 0.20. The results indicate that AC^+^‐related secondary phases appear for ≈0.10 or higher degrees. Incorporating AC^+^ cations has minor effects on the tetragonal‐to‐cubic transition temperature and bandgap energies but significantly reduces the electronic conductivity and *I*–*V* hysteresis. Intriguingly, the results suggest that AC^+^ cations do not strongly interact with the inorganic framework of the studied materials, which might be one of the reasons behind the little stabilization effect of the substituting cations, contrary to general literature reports. This work consolidates the properties of AC_*x*_MA_1−*x*_PbI_3_ materials and contributes to understanding how A‐site cations affect the characteristics of resulting halide perovskites, a topic of an urgent need for better elucidation.^[^ ^26^ ^]^

## 2 Results and Discussion

**Figure 1:**
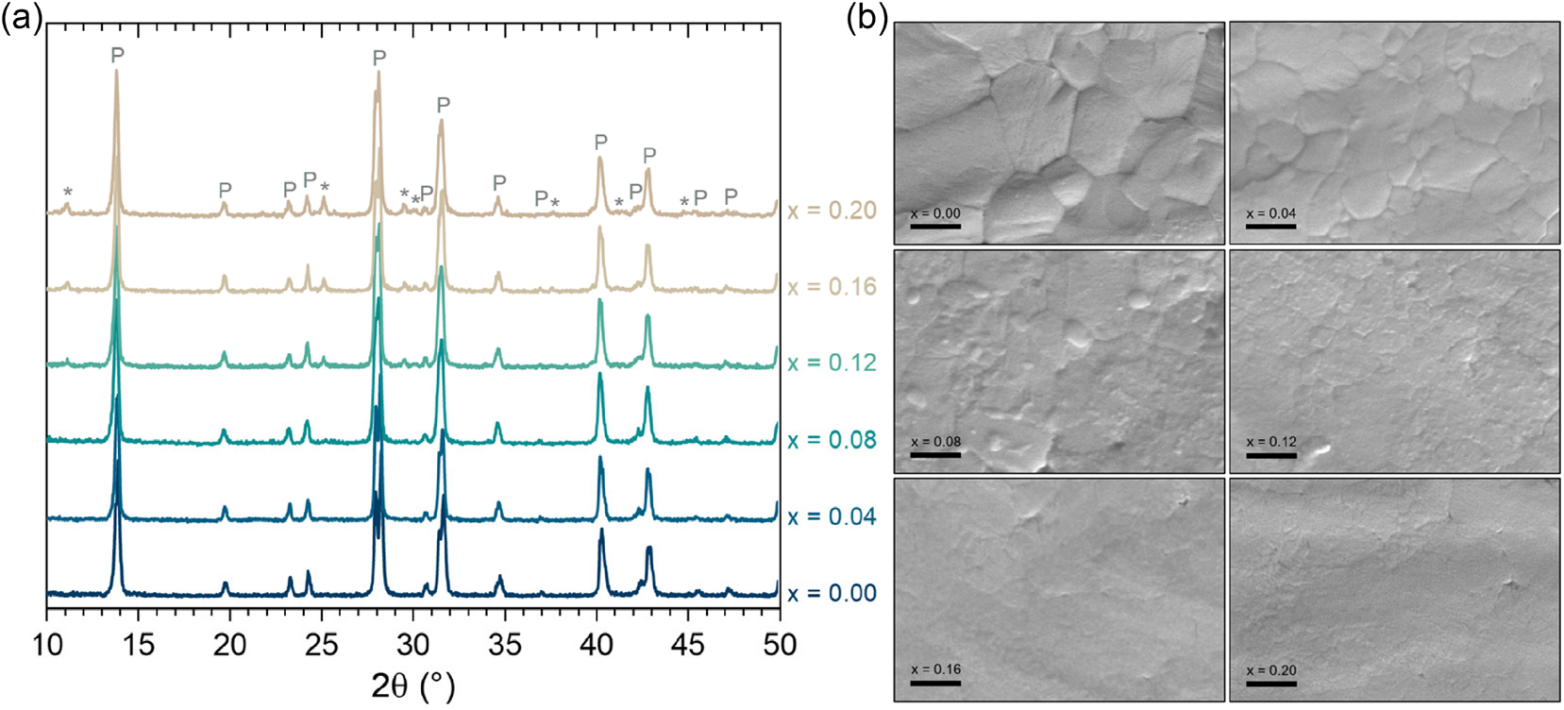
a) XRD patterns of studied AC_*x*_MA_1−*x*_PbI_3_ compositions. Peak assignments are of P: perovskite and *: nonperovskite phases. b) SEM images of the studied compositions. Scale bars are 1 μm.

**Figure** **1**a presents the X‐ray diffraction (XRD) diffraction patterns for all compositions, highlighting the emergence of secondary phases, possibly due to ACPbI_3_ (herein named generally as a nonperovskite phase) segregation, which was identified in the composition with *x* = 0.12. The peaks associated with this phase are labelled by the symbol “*” at ≈11.5°, 25°, 29.9°, 30°, 38°, 41°, and 45°. In all samples, the perovskite phase exhibits tetragonal symmetry, with a possible space group I4 cm.^[^ ^27^ ^]^

As mentioned earlier, a solubility limit in the AC_*x*_MA_1−*x*_PbI_3_ system is reported to be in the wide range from *x* = 0.15 to 0.50, above our findings. We strongly believe that such high values were found because of the synthesizing conditions, materials architecture, and measurement parameters. For example, in one of these studies, the authors used a scanning rate of 10° s^−1^ in XRD measurements, which is too high to obtain precise phase detection. Moreover, using solution‐processed thin films may result in strained conditions with kinetically trapped supersaturated solid solutions where thermodynamic equilibrium cannot be achieved, leading to imprecise solubility limit determination. With prolonged low‐temperature annealing times, our mechanochemical approach can lead to complete reactions (no unreacted PbI_2_ signals are noticeable, e.g., at ≈12.5°), compositional homogeneity, and high crystallinity. In this sense, we are confident that bulk AC_*x*_MA_1−*x*_PbI_3_ perovskites possess a solubility limit of around *x* = 0.11, since the secondary phase appears in the range from *x* = 0.08 to 0.12 and, using the very same mechanochemical synthesis method, a single‐phased AC_0.10_MA_0.90_PbI_3_ composition was obtained.^[^ ^17^ ^]^ Importantly, the use of well‐known mechanochemical methods^[^ ^28^, ^29^ ^]^ guarantees that the final materials have the desired stoichiometry of starting precursors, a feature that may not be secured in solution‐based synthesis, such as of thin films and single crystals. This aspect allows us to establish the obtained solubility limits and acetamidinium variations in the materials with better accuracy. The formation of high crystallinity phases, especially in low AC^+^ contents, can be verified in scanning electron microscope (SEM) micrographs of Figure 1b, where the formation of large grains and well‐defined grain boundaries are evident in the *x* ≤ 0.08 compositions. Increasing AC^+^ content visually leads to smaller grains and less defined grain boundaries, suggesting hindered microstructural development and mitigated ion transport. The disappearance of clear grains coincides with AC^+^ contents above the solubility limit. An intriguing observation, however, is that no distinct microconstituents appear in micrographs, contrary, for example, to GA^+^ contents above the solubility limits in the GA_*x*_MA_1−*x*_PbI_3_ system, where the segregation of GAPbI_3_ is evidenced by the formation of elongated, rod‐shaped phases as its concentration increases.^[^ ^30^, ^31^ ^]^

To better understand AC^+^ incorporation into the perovskite lattice, we analyzed the XRD data of solid solutions in detail. Even though the *x* = 0.12 composition is not single‐phased, it possibly possesses higher solubilized AC^+^ content than *x* = 0.08, depending on the actual solubility limit, which we discussed to be above *x* = 0.10. Even though there is a segregation of ACPbI_3_, the fraction of this phase must be very small and assumed to be insignificant regarding the alteration of the material properties. Therefore, it is assumed that the changes in properties that occur in the analyses in the sequence are due exclusively to the replacement of MA^+^ by AC^+^. Hence, we opted, from this point on, to perform analyses for all compositions from *x* = 0.00 to 0.12.

**Figure 2:**
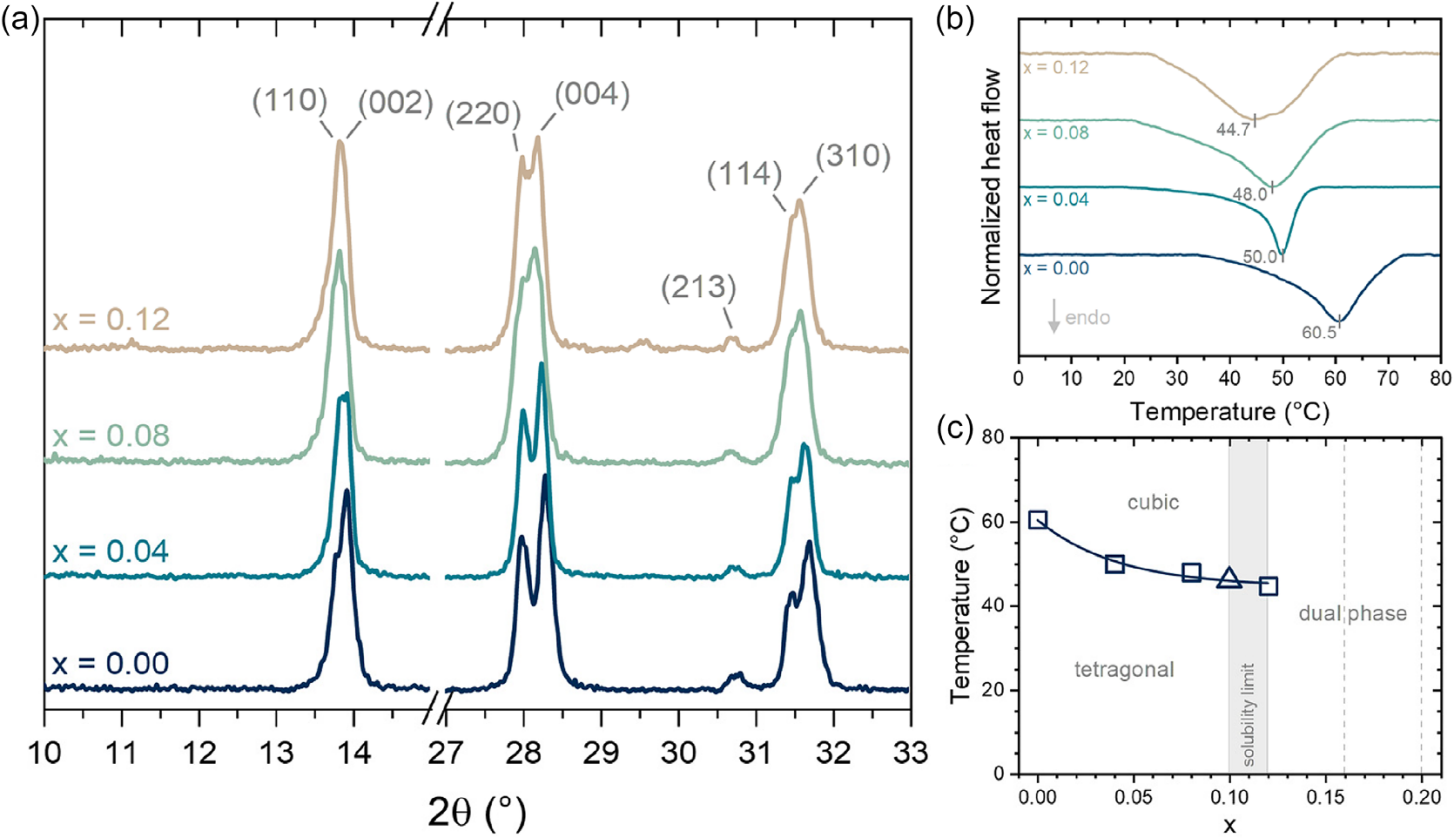
a) XRD patterns of AC_*x*_MA_1−*x*_PbI_3_ solid solution on 2*θ* intervals of main peaks. Indexations are based on the I4 cm space group. b) DSC thermograms obtained on heating regimes. c) The proposed temperature‐composition phase diagram in the studied ranges. The data denoted with a triangle at *x* = 0.10 was taken from.^[^ ^17^ ^]^ The vertical dashed gray lines represent the compositions above the solubility limit, for which the phase transitions were not determined.

**Figure** **2**a displays the main reflection peaks of the perovskite phase, corresponding to the crystallographic planes (110) and (002) near 13.8°, (220) and (004) near 28.0° and 28.3°, and (213), (114), and (310) near 30.7°, 31.4°, and 31.8°, respectively. The intensity of the (213) plane, characteristic of the tetragonal phase, slightly decreases as AC^+^ substitution increases. Additionally, the progressive overlap of peaks (110) with (002), (220) with (004), and (114) with (310) suggests a structural change in the crystal lattice.^[^ ^17^ ^]^ The incorporation of AC^+^ into the structure alters the lattice parameters due to its larger effective ionic radius compared to MA^+^ (respectively, 277 and 217 pm^[^ ^1^ ^]^), leading to the so‐called lattice symmetrization.^[^ ^32^ ^]^ It indicates the stabilization of the cubic phase at lower temperatures, manifesting as a reduction of the tetragonal‐to‐cubic transition (T → C) temperature. This possible T → C trend is corroborated by the thermogram curves obtained from differential scanning calorimetry (DSC), shown in Figure 2b. The transition temperature in the pure MAPbI_3_ sample is ≈60.5 °C (≈334 K), close to the broadly reported value of ≈330 K,^[^ ^33^ ^]^ and progressively decreases with increasing AC^+^ substitution content. Based on the data obtained from XRD and DSC, we propose a temperature‐composition phase diagram of the AC_*x*_MA_1−*x*_PbI_3_ system considering the analyzed ranges in this study, given in Figure 2c.

**Figure 3:**
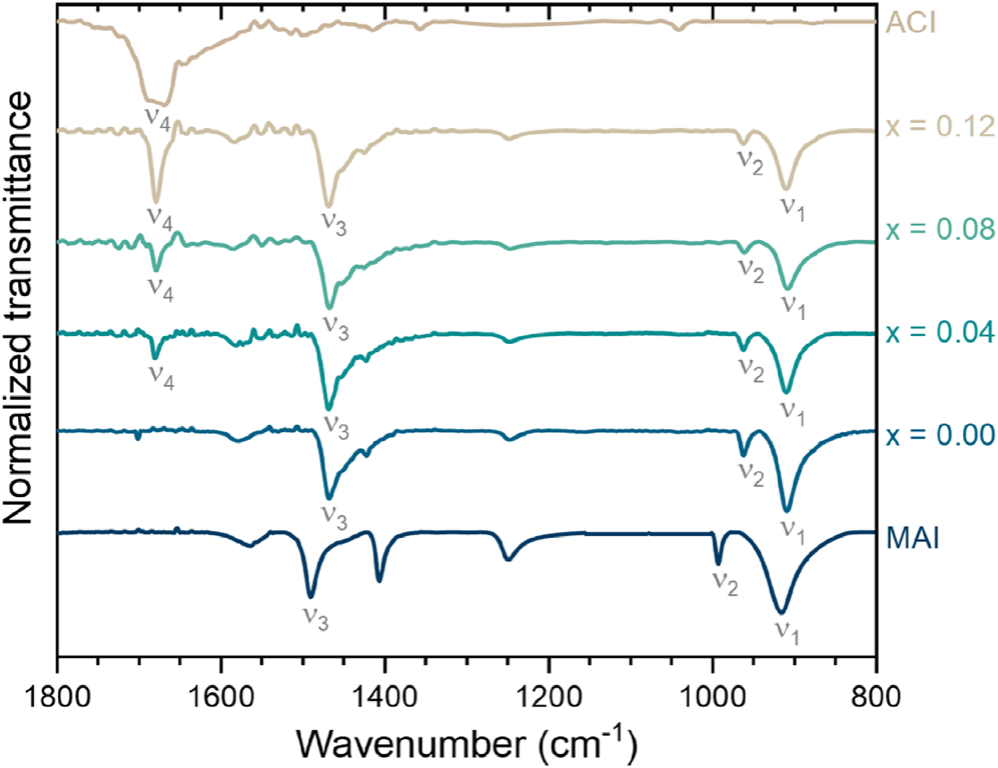
Normalized transmittance spectra obtained by FTIR of the studied AC_*x*_MA_1−*x*_PbI_3_ compositions. The experimental spectra of the MAI and ACI precursors used in this work are also presented for better comparison. The unassigned bands are due to C—C, C—H, and O—H bonds or coupled modes of no analytical use in this work.

To obtain more insights into the interactions of the organic cations at the perovskite lattice, we performed Fourier transform infrared spectroscopy (FTIR) experiments. **Figure** **3** presents the transmittance spectra for all studied compositions, along with the spectra of the precursor materials, MAI and ACI, for reference. Information on the main bands of interest is provided in **Table** **1**. Emphasis is given to the low wavenumber region (700–1800 cm^−1^), where intense bands related to C—N and N—H bonds are detectable. The visual comparison between the spectra shows that all peaks observed in the precursors are also observed in the AC_*x*_MA_1−*x*_PbI_3_ compositions. This is expected as the MAI and ACI peaks are due to vibrations of the MA^+^ and AC^+^ cations, respectively. However, there are considerable shifts in all MA^+^ peaks (modes assigned as $\nu_{1}$, $\nu_{2}$, and $\nu_{3}$) to lower wavenumbers from MAI to the perovskites due to the chemical and structural differences affecting local polarizability and hydrogen bond capabilities.^[^ ^34^ ^]^ On the other hand, the substituent AC^+^ cations on the MA^+^ seem not to affect vibrational modes. It was previously discussed the possible correlation between the dipole moment of the substituting cation on the MA^+^ vibrational dynamics,^[^ ^17^, ^35^ ^]^ where it was hypothesized that substituting cations with much higher (e.g., EA^+^ = 4.97 D) or much lower (e.g., FA^+^ = 0.22 D and GA^+^ = 0) dipole moments than MA^+^ (2.69 D) may provoke displacements of electronic clouds by inductive effects. In this sense, AC^+^ (2.01 D) may not significantly differ from MA^+^ with a similar dipole moment to provoke a detectable shift with the employed experimental conditions.

**Table 1:** Characteristics of assigned bands in FTIR spectra. All values are in cm^−1^. Wavenumber imprecisions are of ±1 cm^−1^. (–): nonexistent bands. Numbers in the first row are the *x* values.

Band	Attribution	Cation	MAI	0.00	0.04	0.08	0.12	ACI
$\nu_{1}$	CH_3_–NH_3_^+^ rocking	MA^+^	914	908	908	907	908	–
$\nu_{2}$	C—N stretching	MA^+^	991	960	961	960	960	–
$\nu_{3}$	NH_3_^+^ sym. bending	MA^+^	1489	1467	1467	1466	1468	–
$\nu_{4}$	C—N stretching	AC^+^	–	–	1680	1678	1678	1678

Regarding the C—N stretching bands, the comparison between AC^+^ and MA^+^ shows that the band on the former occurs at higher wavenumbers, consistent with the order of the C—N bond in AC^+^, which is 1.5, while at MA^+^, it is 1.0. Another expected feature is that the band intensity of C—N stretching (mode $\nu_{4}$) increases with the AC^+^ content. However, an interesting observation is that the position of $\nu_{4}$ does not vary from ACI to AC_*x*_MA_1−*x*_PbI_3_ compositions, contrary to the analogous situation for MA^+^. This suggests that the interaction of AC^+^ cations with its local chemical environment does not significantly differ in the compounds. From information on the fabricant itself, the melting point of ACI is around 100 °C, quite low even compared to MAI, which is around 260 °C. From that perspective, it can be inferred that the lattice energy in ACI is lower than in MAI, which includes the interaction of the organic cation with the inorganic framework. Hence, we can suppose that the AC^+^ bonds with the vicinity in AC_*x*_MA_1−*x*_PbI_3_ are also not particularly strong.

**Figure 4:**
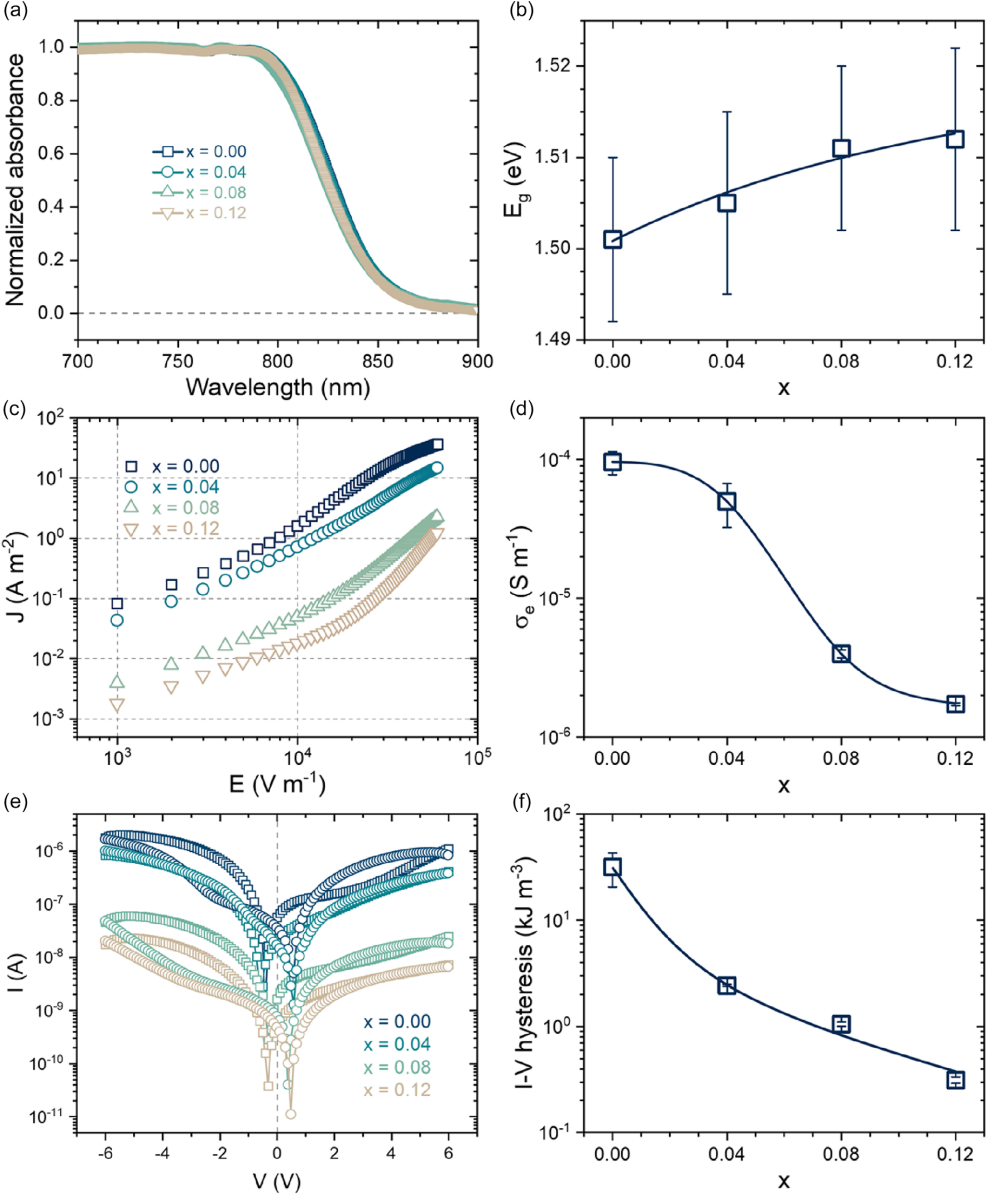
a) Diffuse reflectance UV–Vis absorbance spectra of AC_*x*_MA_1−*x*_PbI_3_ solid solutions and b) corresponding band gap energies. Error bars are the fit residuals. c) Forward *J*–*E* data and d) electronic conductivity determined at low electric fields. e) Up and down *I*–*V* data and f) extracted *I*–*V* hysteresis. In (c) and (e), the curves shown are a mean of four measurements. The original data are given in Supporting Note 3, Supporting Information. Errors bars in (d) and (f) are the standard deviation from the mean. Blue continuous lines in *x*‐dependent graphs are guides to the eyes.

As materials are known for electrochemical and optoelectronic applications, addressing the AC^+^ effects on the electrical properties of resulting materials is crucial. **Figure** **4**a presents the UV–Vis absorbance curves, showing intense and steep absorption in a narrow range from 850 to 800 nm for all compositions, suggesting AC^+^‐independent bandgap energies ($E_{g}$). Based on these spectra, the $E_{g}$ values were estimated through a linear fit of data dispersed in a Tauc plot using the Kubelka–Munk‐modified Tauc equation, given by ${\left(F(R).h\nu \right)}^{1/\gamma }=B(h\nu -E_{g})$, where hν is the incident photon energy, *γ* a factor that depends on the nature (direct or indirect) of the electronic transition, and F(R) is the Kubelka–Munk function, given by $F(R)={\left(1-R\right)}^{2}/2R$, where *R* is the reflectance of an infinitely thick sample.^[^ ^36^, ^37^ ^]^ In our analysis, we considered electronic transitions related to a direct bandgap (γ=1/2), as reported for halide perovskites.^[^ ^38^ ^]^ The corresponding linear fits are provided in Supporting Note 1, Supporting Information. The estimated bandgap values are shown in Figure 4b. As expected, the bandgap values show marginal changes within the 1.50 to 1.51 eV interval, with a subtly tendency to increase with the AC^+^ content. This behavior is the most common for MAPbI_3_‐based solid solutions, with FA‐rich compositions being the few exceptions.^[^ ^39^, ^40^ ^]^ In general, band gap energies in halide perovskites are not very sensitive to the composition of the A‐site, because the valence and conduction bands are formed mainly by the outer B and X orbitals, with the cations of the A‐site being merely indirect effects.^[^ ^41^ ^]^

To evaluate possible changes in charge carrier transport behavior with composition, current–voltage measurements were performed under a fast scan condition to distinguish electronic charge carrier transport from its ionic counterparts. Figure 4c presents forward current density (J=I/A) versus applied electric field (E=V/d) curves, where *A* and *d* are, respectively, the electrode area (≈8.10^−7^ m^2^) and sample thickness (≈500 μm). Cross‐section images are given in Supporting Note 2, Supporting Information. For most of the measured electric field range, predominantly at low values, the dominant electrical conduction regimes are ohmic for all samples (slope equal to 1 in log–log scale).^[^ ^42^ ^]^ At higher fields, the curves appear to transition to nonohmic regimes, with general behavior following a $J\propto V^{n}$ function. Even though several models were applied to study in detail the conduction mechanisms in halide perovskites,^[^ ^40^, ^43^, ^44^, ^45^, ^46^ ^]^ we simplified our analysis by restricting it to the ohmic range. Having in mind that $J=\sigma_{e}E$, the electronic conductivities ($\sigma_{e}$) of all compositions were estimated with a simple linear fit. The results are shown in Figure 4d, where we observe that incorporating the AC^+^ cation affects significantly the material's electrical conductivity. For the pure composition, the conductivity is 10^−4^ S m^−1^, whereas at the solubility limit, it decreases by nearly two orders of magnitude (approaching 10^−6^ S m^−1^). Since $\sigma_{e}=qn_{e}\mu_{e}$, where $n_{e}$ and $\mu_{e}$ are, respectively, the electronic carrier density and mobility, and knowing that in A‐site substituted MAPbI_3_, only small $n_{e}$ changes are expected,^[^ ^17^, ^39^ ^]^ it can be concluded that electronic mobility is the main factor responsible for these differences. From the general perspective, solid solutions present higher carrier scattering due to inherent lattice heterogeneity. From the particular perspective, it seems that AC^+^ cations are more efficient than other substituent organic cations in reducing the electronic carrier mobility,^[^ ^17^, ^47^ ^]^ proposedly due to a combination of its large size, considerably high dipole moment, and presence of π electrons. It was recently found that AC_0.1_MA_0.9_PbI_3_ thin films have a sixfold lower carrier mobility than their unsubstituted counterpart.^[^ ^48^ ^]^ In this sense, the expected behavior is electronic conductivity reduction with the AC^+^ content.

Another important charge transport characteristic in halide perovskites is the well‐known *I*–*V* hysteresis, often associated with perovskite solar cells.^[^ ^49^ ^]^ To evaluate it in our AC_*x*_MA_1−*x*_PbI_3_ compositions, we used the same methodology employed in other works,^[^ ^17^, ^30^, ^40^, ^47^, ^50^ ^]^ where the power losses that occur in current–voltage cycles are measured in up and down regimes under low scan rates. Representative curves for each composition and the determined *I*–*V* hysteresis values are shown, respectively, in Figure 4e,f. Notably, increasing AC^+^ contents provoke significant hysteresis reduction, which may be one explanation for AC^+^‐containing solar cells having better operational stability than unsubstituted compositions. Knowing the correlations between the *I*–*V* hysteresis phenomenon and mobile ions in halide perovskites,^[^ ^51^, ^52^, ^53^ ^]^ these results suggest that AC^+^ effectively mitigate ion transport, as already reported.^[^ ^17^, ^22^, ^54^ ^]^ Considering that, the obvious correlation between higher AC^+^ content and lower *I*–*V* hysteresis is unsurprising.

**Figure 5:**
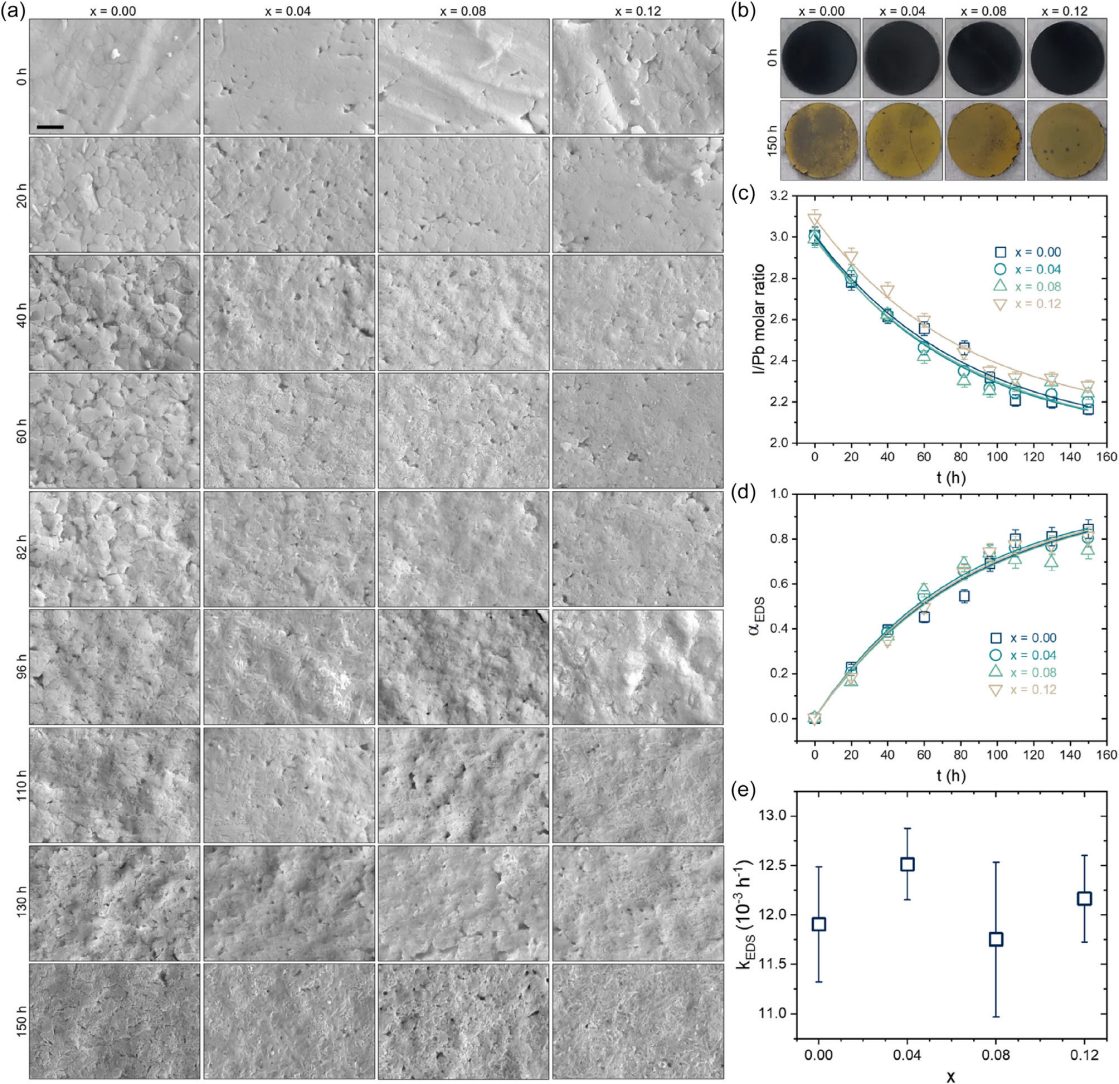
a) SEM micrographs of the AC_*x*_MA_1−*x*_PbI_3_ samples obtained from SEM measurements as a function of the light treatment time. The scale bar of 2 μm is the same for all SEM images. b) Photographs of the samples taken before and after the light‐accelerated degradation test. c) I/Pb molar ratio as a function of the treatment time. d) Degradation extent as a function of the time treatment. Lines are the fits with a first‐order reaction kinetic model. e) The degradation kinetic constant is calculated for each substitution degree. Error bars are the fit residuals. Lines in (c) are reconstructed curves using the results from the fits of (d).

One of the main issues with halide perovskites is their tendency to degrade under several environmental conditions, as discussed earlier. To investigate the effects of AC^+^ cation substitution on the stability and degradation behavior of AC_*x*_MA_1−*x*_PbI_3_ materials, we performed light‐accelerated degradation and characterized the transformations with analytical techniques. **Figure** **5** presents the results of these tests, obtained through SEM and energy‐dispersive X‐ray spectroscopy (EDS) analyses. Figure 5a displays micrographs of the different compositions as a function of light treatment time at temperatures ranging from 30 to 35 °C. In general, as treatment time increases across all compositions, micrographs reveal progressive changes, from regular microstructures with defined grains to a laminated aspect with visible pores and voids, very similar to microstructural evolution under thermal treatments of.^[^ ^47^, ^55^ ^]^ These changes can possibly be due to mass loss of volatiles, as it is known that when MAPbI_3_‐based materials are exposed to light and oxygen, gaseous products such as CH_3_NH_2_, I_2_, and HI can be formed, leaving solid, yellow PbI_2_.^[^ ^56^, ^57^, ^58^ ^]^ It is corroborated by the fact that the initial and final aspects of samples, as shown in Figure 5b, change from black to yellow, indicating that all samples undergo severe degradation after 150 h of illumination. The fact that there are no clear differences between the compositions, from both visual and microstructural perspectives, suggests that AC^+^ incorporation does not significantly alter the materials’ stability against the imposed conditions. To quantify the influence of AC^+^ substitution on the stability, we used quantitative EDS analyses to estimate the samples’ I/Pb molar ratio over time. Figure 5c shows the expected decaying rates of the I/Pb ratio, starting from ≈3 of AC_*x*_MA_1−*x*_PbI_3_ pristine perovskites and approaching ≈2 of PbI_2_, a behavior verified for all compositions. To obtain the degradation rates, we converted the time‐dependent I/Pb ratios ($R_{t}$) to a degradation extent ($\alpha_{\text{EDS}}$) using the relation $\alpha_{\text{EDS}}=R_{0}-R_{t}$, where $R_{0}$ is the initial I/Pb ratio (theoretically equal to three). Then, the experimental data was fitted with a first‐order reaction kinetic model, as employed elsewhere,^[^ ^17^, ^39^ ^]^ given by $\alpha_{\text{EDS}}=1-\text{exp(}-k_{\text{EDS}}t)$, where $k_{\text{EDS}}$ is the degradation kinetic constant obtained from EDS measurements. The fitted data and $k_{\text{EDS}}$ of each composition are given, respectively, in Figure 5d,e. Results show that the time‐dependent degradation extent is essentially not dependent on the AC^+^ content, which is quantitatively verified from statistically equivalent $k_{\text{EDS}}$ values based on the error bars.

**Figure 6:**
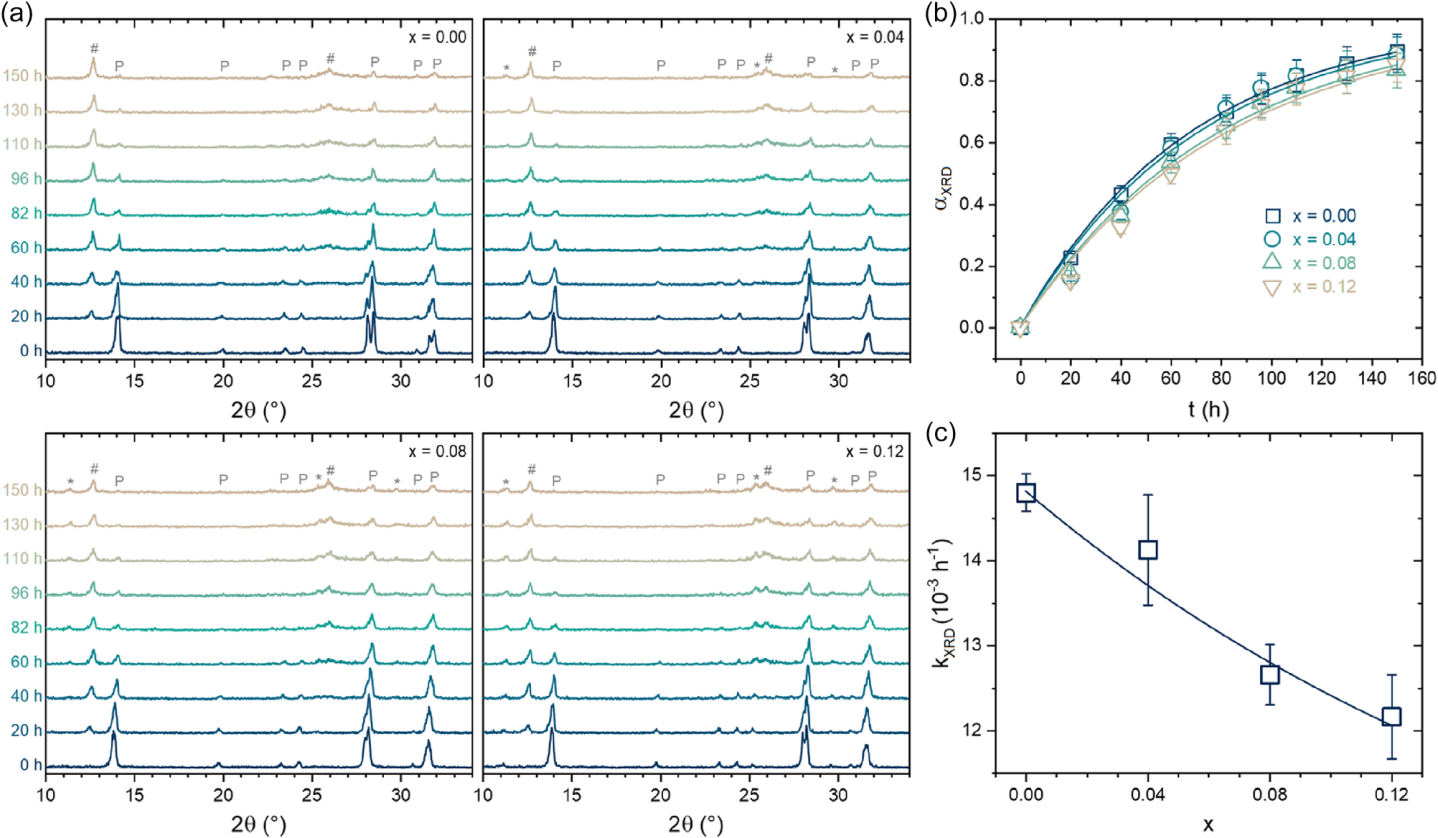
a) XRD data of the AC_*x*_MA_1−*x*_PbI_3_ samples submitted to different light‐accelerated degradation times. Peaks assignments are of P: perovskite, *: nonperovskite, and (#) PbI_2_ phases. b) Degradation extent as a function of the time of treatment. Lines are the fits with a first‐order reaction kinetic model. c) Obtained degradation kinetic constants. Error bars are the fit residuals.

Additional XRD measurements on samples under the same degradation conditions were conducted to validate the previous findings further. These results are summarized in **Figure** **6**. The time‐dependent diffraction patterns shown in Figure 6a for each composition reveal peaks corresponding to the perovskite phase (P), the PbI_2_ phase (#), and nonperovskite phases (*). Notably, peaks of PbI_2_ can already be detected right after the first measurement (20 h of light exposure), evidencing early signs of perovskite degradation, consistent with the previous SEM/EDS results. Moreover, as expected, the intensity of perovskite peaks decreases continuously, making clear the volatilization of organic moieties and leaving PbI_2_, whose peaks increase with time.

For all samples, regardless of the AC^+^ content, the perovskite peaks almost vanish after 150 h, showing nearly complete decomposition. It indicates that AC^+^ substitution does not lead to a significant qualitative change in the XRD patterns. Simultaneously, the AC^+^‐containing materials display the later formation of peaks of the nonperovskite phase. This phenomenon has already been verified in other mixed A‐site systems composed of cations with low solubility limits, such as acetamidinium itself, guanidinium, dimethylammonium, and imidazolium.^[^ ^17^, ^47^, ^55^, ^59^ ^]^ We believe this is due to an oversaturation of nonvolatized AC^+^ cations in the remaining perovskite lattice. On the other hand, it is apparent that, after the test, the ACPbI_3_ nonperovskite peaks have the same intensity regardless of the AC^+^‐containing composition, suggesting that AC^+^‐related molecules may also be volatilized. To support this hypothesis, we rescued the idea that FTIR results indicated that AC^+^ cations are not particularly bound to the inorganic framework.

Finally, to quantitatively access the degradation kinetics with XRD data, the sum of areas (integrated intensities) of perovskite peaks at each time ($A_{t}$) was determined and converted to the degradation extent ($\alpha_{\text{XRD}}$) using the relation $\alpha_{\text{XRD}}=1-A_{t}/A_{0}$, where $A_{0}$ is the sum of areas at the beginning of the experiment. The time‐dependent degradation extent results shown in Figure 6b are visually the same as the obtained through EDS experiments and were analogously fitted with a first‐order reaction kinetic model, given by $\alpha_{\text{XRD}}=1-\text{exp(}-k_{\text{XRD}}t)$, where $k_{\text{XRD}}$ is the degradation kinetic constant obtained with the XRD data. The resulting composition‐dependent $k_{\text{XRD}}$ values are given in Figure 6c. From these results, a few conclusions can be drawn. First, the absolute values obtained from the two methods are quite similar, in the 12–15.10^−3^ h^−1^ range, indicating that the perovskite phase degradation occurs by volatilizing organic compounds from the samples. Second, in the XRD case, there seems to be a trend of increasing stability ($k_{\text{XRD}}$ decreasing) with the AC^+^ content, we must consider comparing the reported values using the same methodology. Considering that the degradation kinetic constant was also close to 15.10^−3 ^h^−1^^[^ ^17^, ^39^ ^]^ for MAPbI_3_, we attest the good reproducibility of this light‐accelerated degradation test. By comparing the present values of others reported in these two references, where, depending on the composition, the degradation kinetic constants may drop by more than a fivefold fashion, it turns that the effects of AC^+^ cations are effectively minor. In this regard, we believe that eventual chemical stability increase observed in the literature upon substitution with AC^+^ might be true only in devices and in controlled conditions, such as under inert atmospheres, in thin films, and with the additional layers present in devices. The results here obtained in bulk crystals show that using AC^+^ does not lead to inherently higher chemical stabilities.

## 3 Conclusions

The structure, electrical properties, and chemical stability of AC_*x*_MA_1−*x*_PbI_3_ perovskites were investigated using a combination of characterization techniques as investigation tools. The determined solubility limit of AC^+^ cations in the MAPbI_3_ lattice was only about *x* = 0.10, much lower than currently reported values in the literature. All single‐phased AC_*x*_MA_1−*x*_PbI_3_ compositions are tetragonal at room temperature, slightly decreasing the tetragonal‐to‐cubic phase transition temperature upon substitution with AC^+^. Contrary to the literature's proposals, our results suggest that the AC^+^ cations are not strongly bound to the inorganic framework and do not lead to significant stability against light‐accelerated degradation. On the other hand, corroborating the literature, AC^+^ cations effectively mitigate charge transport and *I*–*V* hysteresis, which may be the main reason behind the reported increase in solar cell efficiencies and operational stability in AC^+^‐containing halide perovskites. Our results consolidate the effects of this common organic cation and broaden the knowledge of composition–property correlations in mixed A‐site halide perovskites.

## 4 Experimental Section

For this research, pellets of six halide perovskite compositions of the AC_*x*_MA_1−*x*_PbI_3_ system were mechanochemically synthesized, with AC^+^ contents of *x* = 0.00, 0.04, 0.08, 0.12, 0.16, and 0.20. The precursors used were methylammonium iodide (MAI, Sigma–Aldrich, 99%), lead iodide (PbI_2_, Sigma–Aldrich, 99%), and acetamidinium iodide (ACI, Sigma–Aldrich, 98%). The mechanochemical process involved mixing a total of about 1 g of precursors, in the desired proportions, in a polytetrafluoroethylene container with small zirconia spheres, followed by gently stirring for ≈40 min. This resulted in a black powder, which was then sieved through a 250 μm mesh, totaling about 0.9 g for each composition. Subsequently, pellets with a mass of ≈150 mg and a diameter of 10 mm were formed using a metal mold and compacted at a pressure of 250 MPa. Finally, the pellets were sintered in a furnace with a heating rate of 5 °C min^−1^ up to 120 °C with isotherm for 2 h, followed by a slow cooling to room temperature.

To analyze the crystal structure, XRD was performed using a Rigaku diffractometer, model Ultima IV, with Cu K_α1_ radiation (*λ* = 1.5406 Å), operating at 40 kV and 40 mA, in step scan mode with a scanning step of 0.02° (4 s integrating time) over a 2*θ* range of 10° to 50°. To obtain the micrographs of the samples, a Zeiss SEM model EVO LS15 was used, operating with a tungsten beam in the voltage range of 10 to 20 kV, with secondary electron analysis (SE mode). Coupled EDS analyses were performed using Oxford Instruments INCA x‐act. Iodine and lead elemental composition estimations were based on EDS analyses in regions of 1278 × 684 μm. FTIR was carried out on a Nicolet NEXUS 670, with 256 scans, a resolution of 2, in a wavenumber range of 800 to 1800 cm^−1^, using the transmission mode of pellets containing ≈2 mg of perovskite powder dispersed in ≈200 mg of KBr. DSC thermograms were acquired using 10–12 mg fragments of sintered pellets in hermetic aluminum crucibles with a DSC 25 apparatus (TA Instruments). Measurements were performed in a temperature range of −50 to 100 °C with a heating rate of 5 °C min^−1^ under a vacuum atmosphere. Diffuse UV–Vis reflectance spectroscopy measurements were collected using a Shimadzu UV‐2600 spectrophotometer, with a range of 200–1400 nm, in low‐speed mode with a data step of 1 nm. For electrical measurements, nine circular gold electrodes of ≈1 mm in diameter (photos given in Supporting Note 4, Supporting Information) were sputtered onto the top of the perovskite wafers, while a gold electrode was deposited over the entire bottom surface of the sample. Steady‐state current–voltage (*I*–*V*) data were collected in four randomly chosen electrodes using a Keithley 6517B electrometer in the dark at room temperature. Up–down cycle measurements were performed from ±6 V at a scan rate of 0.1 V s^−1^, while forward measurements were conducted from 0 to +30 V at a scan rate of 0.5 V s^−1^. Unless otherwise stated, all measurements were performed at room temperature (≈25 °C).

A custom‐built setup was developed for the light‐accelerated degradation test, consisting of a wooden box with an interior featuring a rotation system and a lamp. The samples were evenly distributed in a Petri dish, positioned at the center of the rotating platform (6 rpm clockwise), and subjected to direct and mirror‐reflected illumination from a LED lamp. The lamp specifications were 6 W power, 560 lm luminous flux, and a cool white color (6500 K). The box, measuring 30 × 20 × 15 cm, remained closed and sealed with adhesive tape throughout the test. Internal humidity was kept as low as possible using silica beads, and temperature and relative humidity were continuously monitored. This is the same setup used in earlier studies.^[^ ^17^, ^39^ ^]^ After periods of up to 150 h, samples were removed from the setup. Samples of each composition were used for SEM/EDS measurements using the same equipment and conditions mentioned earlier. Simultaneously, a defined composition sample was selected over time for XRD measurements. In this case, the same equipment before was used, but under 30 kV and 20 mA, in continuous scan mode with a scanning rate of 0.02° s^−1^ over a 2*θ* range of 10° to 35°.

## Supporting Information

The supporting information file contains a photo of the deposited electrodes, Tauc plots with linear fits used to determine bandgap energies, cross‐section SEM images of samples used for electrical measurements, and the complete set of current–voltage data used to estimate electronic conductivities and *I*–*V* hysteresis.

## Conflict of Interest

The authors declare no conflict to interest.

## Author Contributions

**Fernando Brondani Minussi**: conceptualization (leading); data curation (leading); formal analysis (leading); investigation (leading); methodology (leading); validation (leading); visualization (leading); writing—original draft (leading); writing—review and editing (leading). **Rafaela Coutinho de Oliveira Santos**: conceptualization (support); data curation (support); formal analysis (support); investigation (support); methodology (support); writing—original draft (support); writing—review and editing (support). **Marco Antonio de Mello Teixeira**: conceptualization (support); data curation (support); formal analysis (support); investigation (support); methodology (support); writing—original draft (support); writing—review and editing (support). **Rogério Marcos Silva Jr.**: investigation (support); methodology (support); writing—original draft (support); writing—review and editing (support). **Eudes Borges Araújo**: conceptualization (support); methodology (support); resources (leading); writing—original draft (support); writing—review and editing (support); supervision (leading); project administration (leading); funding acquisition (leading).

## Supporting information

Supplementary Material

## Data Availability

All data that support the findings of this study are contained in the main text or Supporting Information.
