# Supplementary material for: An Exploration of the Role of Acetamidinium Substitution in Methylammonium Lead Iodide Perovskites
Source: Chemphyschem. 2025 Nov 23;27(1):e202500259. doi: 10.1002/cphc.202500259 (PMC12810660; doi:10.1002/cphc.202500259)
Supplement: Supplementary file 1 — Supplementary Material [file CPHC-27-e202500259-s001.pdf]

SUPPORTING INFORMATION

An exploration of the role of acetamidinium substitution in methylammonium lead iodide perovskites

F. B. Minussi[1*], R. C. O. Santos[1], M. A. M. Teixeira[1], R. M. Silva Jr[2], E. B. Araújo[1]

[1] Department of Physics and Chemistry, São Paulo State University, 15385-000, Ilha Solteira, SP - Brazil

[2] Department of Electrical Engeneeering, São Paulo State University, 15385-000, Ilha Solteira, SP - Brazil

CONTENTS

Supplementary Note 1: Tauc plots for determination of band gap energies ________________ 1

Supplementary Note 2: Thickness of samples ____ 2

Supplementary Note 3: Current-voltage data ____ 3

Supplementary Note 4: Electrode configurations ______________________________________ 4

[*] corresponding author: f.minussi@unesp.br

Supplementary Note 1: Tauc plots for determination of band gap energies

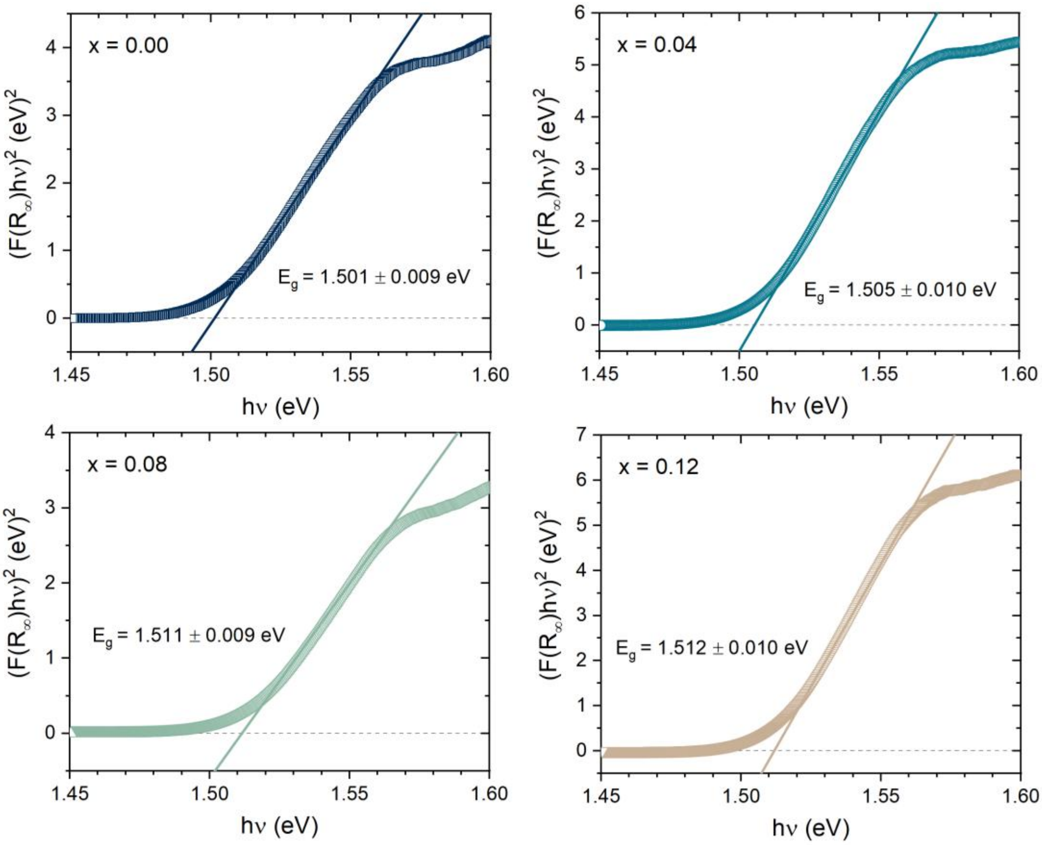

Figure S1 - Tauc plots and respective linear fits of the $AC_xMA_{1-x}PbI_3$ samples.

Supplementary Note 2: Thickness of samples

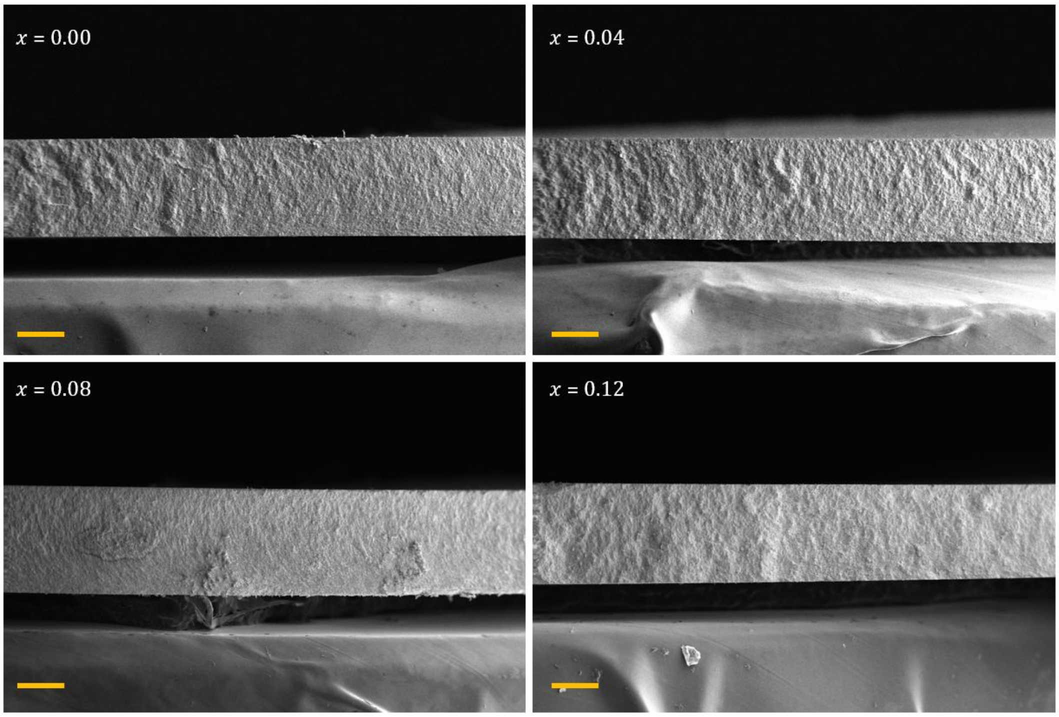

Figure S2 - Cross-section SEM images of the $AC_xMA_{1-x}PbI_3$ samples used in the electrical characterizations. Scale bars are of 200 μm.

Supplementary Note 3: Current-voltage data

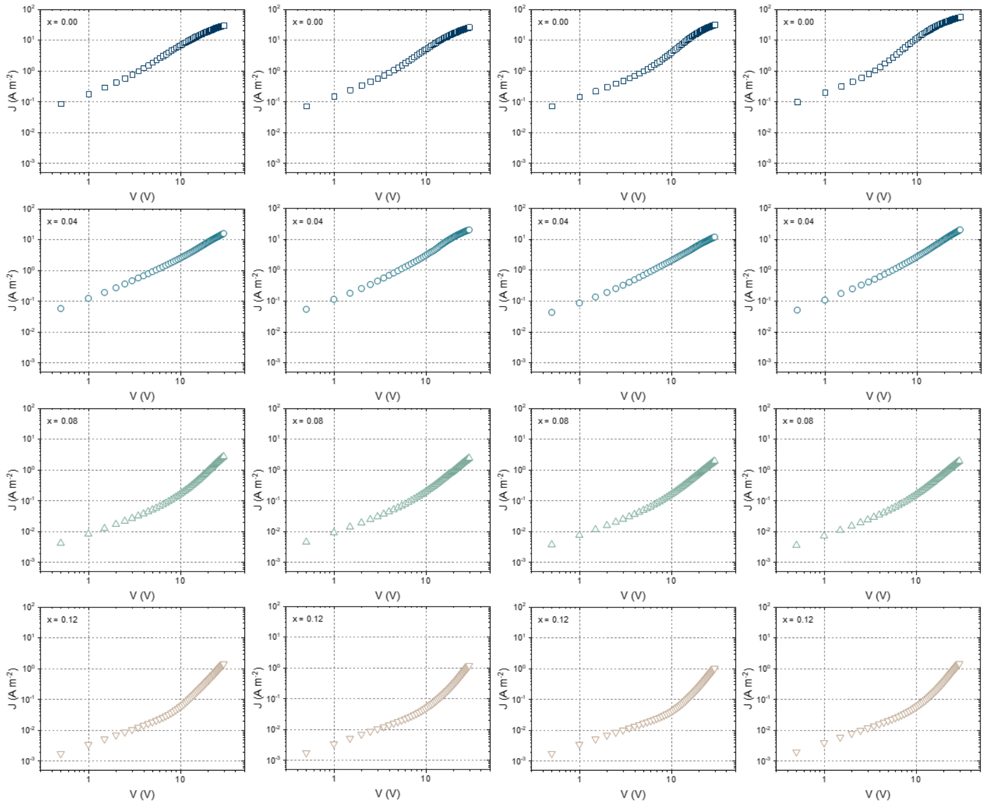

Figure S3 - Four forward measurements with a scan rate of 0.5 V s^{-1} used to evaluate the electronic conductivity in samples of each $AC_xMA_{1-x}PbI_3$ composition.

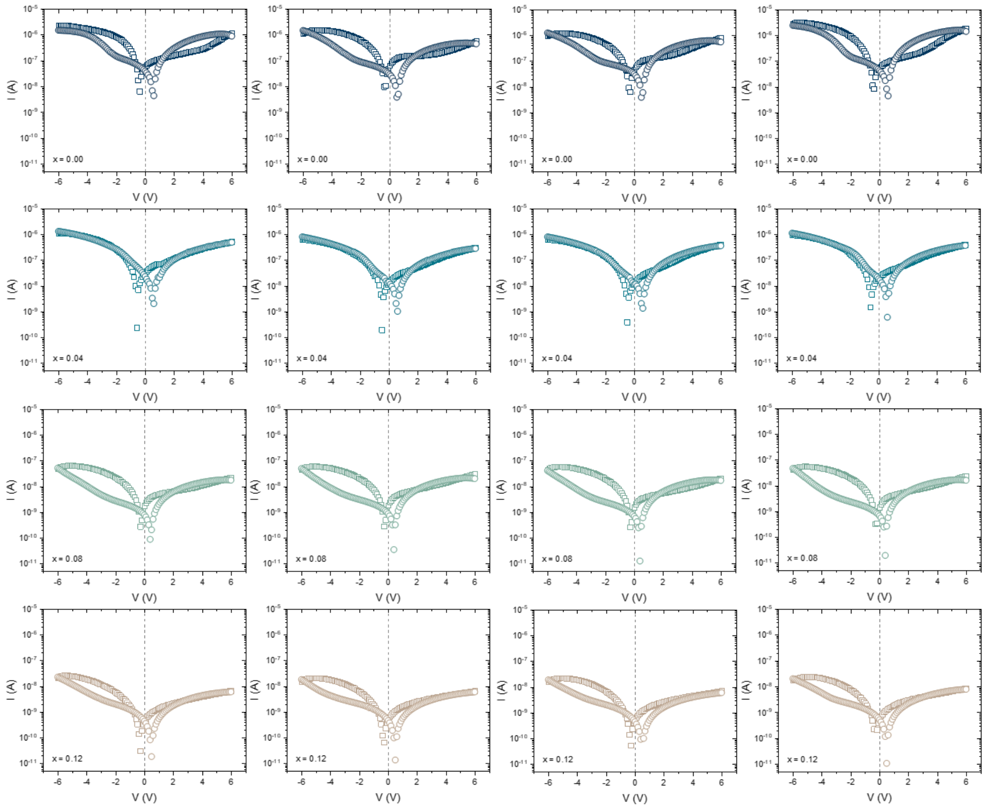

Figure S4 - Four measurements of up (squares) and down (circles) cycles with a scan rate of 0.1 V s^{-1} used to evaluate the *I*-*V* hysteresis.

Supplementary Note 4: Electrode configurations

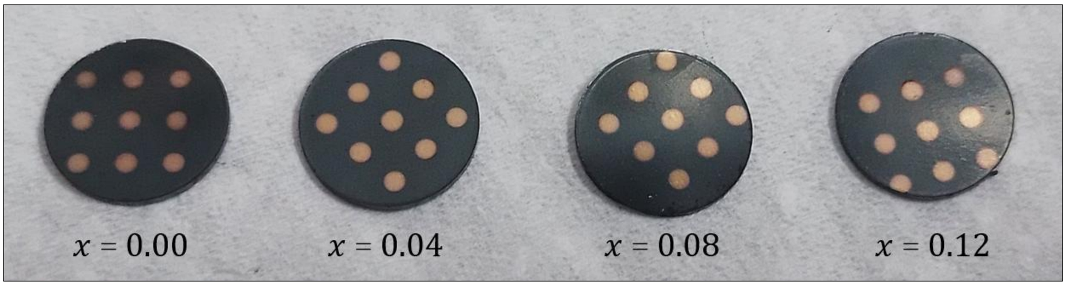

Figure S5 - Samples of $AC_xMA_{1-x}PbI_3$ with deposited electrodes.
